# Low Reward and Heightened Threat Neural Activity are Both Associated with Heightened Anhedonia: A Transdiagnostic Perspective

**DOI:** 10.21203/rs.3.rs-9955077/v1

**Published:** 2026-07-03

**Authors:** Robin Nusslock, Richard E. Zinbarg, Katherine S. Young, Ann L. Carroll, Iris K.Y. Chat, Cody A. Cushing, Michelle G. Craske

**Affiliations:** 1 Department of Psychology, Northwestern University,; 2 Institute for Policy Research, Northwestern University,; 3 The Family Institute at Northwestern University,; 4 Department of Psychology, University of California, Los Angeles,; 5 Department of Psychiatry & Biobehavioral Sciences, University of California, Los Angeles

## Abstract

Psychiatric neuroscience typically examines categorical diagnoses and brain systems in isolation. This approach is complicated by comorbidity and within-diagnosis heterogeneity and fails to evaluate whether relationships are unique to, or common across, neural circuits. To address this, we examined associations between reward and threat neural activity and symptoms of General Distress, Fears, and Anhedonia-Apprehension from the tri-level model of depression and anxiety. Data were collected on 339 participants aged 18–19; 272 completed a monetary incentive delay task (MID) assessing reward neural activity and a Pavlovian fear learning task assessing threat neural activity. We used full-information maximum likelihood to handle missing data and thus retained all 339 participants in each analysis. As predicted, heightened Anhedonia-Apprehension was associated with lower orbitofrontal cortex activity during reward receipt in the MID task (b = −.34, se = .147, p = .021). This relationship was specific to Anhedonia-Apprehension relative to other symptom dimensions and to orbitofrontal activity during reward receipt relative to threat-related neural activity. Heightened Anhedonia-Apprehension was also associated with lower ventral striatal activation during reward anticipation in the MID reward task (b = −.53, se = .218, p = .015). Unexpectedly, heightened Anhedonia-Apprehension was also associated with heightened threat neural activity during the extinction phase of the Pavlovian fear learning task (b = .21, se = .066, p = .001). Findings highlight the importance of examining associations between specific brain systems and empirically derived symptom dimensions to better understand mental health problems and develop targeted interventions.

## Introduction

Understanding the neural bases of appetitive and defensive responding that guide approach toward reward and avoidance of danger or punishment can inform targeted treatments for depression and anxiety (1). Substantial progress has been made in elucidating disruptions in reward- and threat-related neural functioning in these conditions. Yet, without evaluating both reward and threat activity within the same individuals, the specificity of effects remains unknown. This study examines relationships between reward and threat neural activity with symptoms common across anxiety and depression, versus symptoms more specific to depression versus anxiety.

The corticobasal ganglia neural circuit is involved in reward processing and approach motivation (2,3). Within the basal ganglia, the ventral striatum anticipates rewards, whereas the pallidum and putamen are involved in reward-incentive learning and behaviors (2,4,5). Medial portions of the orbitofrontal cortex (OFC) code the subjective experience of pleasures, assess the probability rewards, and guide reward-based learning and decision making (3).

Depression is associated with reduced activation in the ventral striatum, pallidum, and putamen to rewarding stimuli, reflecting reduced motivation to pursue rewards and anhedonia (6–8). There are some inconsistencies in findings on OFC activation in depression, with some studies reporting heightened medial OFC responses to rewards and others reporting lower activation in depression (6,7,9). However, growing evidence suggests anhedonia in depression is associated with lower medial OFC activation, reflecting blunted reward sensitivity (10). In either case, disrupted OFC activation is likely implicated in maladaptive regulation of subcortical reward processing in the basal ganglia (7,11,12).

A common paradigm for assessing threat responding is Pavlovian fear conditioning and extinction. The amygdala is implicated in fear acquisition and immediate threat responses, though human fear conditioning studies often fail to detect greater amygdala activation to the conditioned stimulus, perhaps due to low threat salience (13). The bed nucleus of the stria terminalis (BNST) supports extended fear states, while the anterior insula supports visceral and emotional functions (13–15). Fear extinction involves the hippocampus, which contextualizes extinction memories, the dorsal anterior cingulate cortex (dACC), involved in action execution and emotion regulation (16), and the ventromedial prefrontal cortex (vmPFC), involved in top-down regulation of brain systems involved in fear generation (2,6,7).

Neurobiological models of anxiety disorders posit heightened amygdala, BNST, and anterior insula activation to threat cues during fear acquisition, reflecting heightened threat generating brain activity; lower dACC and vmPFC activation during extinction and extinction retrieval, reflecting reduced emotion regulation and inhibition of threat responses; and lower hippocampal activation, potentially underlying fear generalization (19,20). However, evidence is mixed. Some studies report associations between amygdala and anterior insula activation with anxiety (21,22), but others do not (19,23). Further, there are inconsistencies regarding which phase of conditioning and extinction (i.e., acquisition, extinction, extinction recall) is most associated with lower vmPFC activation in anxiety disorders (19,24).

Most studies have compared neurocognitive functions in diagnostic groups versus healthy controls, with findings complicated by comorbidity and within-diagnosis heterogeneity. The tri-level model is a dimensional framework comprising three factors corresponding to symptoms that are shared (General Distress) and symptoms relatively specific to depression (Anhedonia-Apprehension) and anxiety (Fears) (25–27). General Distress characterizes negative emotions such as anxiety and irritability common to anxiety and depression (25). Anhedonia-Apprehension reflects low positive affect and decreased approach motivation (25) and Fears captures variance common to agoraphobic, interoceptive, social and specific phobia symptoms (25). This model is replicable (25–27) and shares features with the HiTOP Internalizing Spectrum (28).

The current study evaluates cross-sectional associations between reward and threat neural activity, measured using two tasks within the same participants, and the broad factors of the tri-level model of depression and anxiety. This allows us to test whether neural correlates are specific versus common to reward and threat processes and specific versus common to broad symptom dimensions across depression and anxiety. To distinguish effects attributable to shared versus unique variance across regions-of-interest (ROIs), we use a latent construct approach where justified by the data. Associations between latent variables and tri-level factors reflect effects attributable to shared variance among the ROIs, whereas the portion of an ROI independent of the latent variable reflects effects attributable to that ROI’s unique variance. We examine relationships between tri-level symptom factors and both reward and threat latent variables, as well as variance unique to each brain region (i.e., ROI) within each reward and threat latent variable. Participants were aged 18–19 years, a period characterized by key brain development (29) and heightened risk for internalizing psychopathology (30).

Identifying shared and unique brain systems that are specific to, versus common across, symptom dimensions may help resolve inconsistencies in research on depression and anxiety. We hypothesized that higher Anhedonia-Apprehension would be uniquely associated with lower reward-generating (ventral striatum, pallidum, putamen) and regulating (OFC) brain activity during the monetary incentive delay (MID) task (31). We further predicted that General Distress would be less related to reward-related brain activity than Anhedonia-Apprehension. To test for specificity, we examined whether these associations (a) remain when the model includes General Distress and Fears (b) remain when accounting for threat-related neural activity (c) are stronger than the associations between Anhedonia-Apprehension with neural response to threat, and (d) are specific to reward anticipation or outcome.

We also hypothesized that General Distress and Fears would relate to heightened threat-generating brain activity (amygdala, BNST, anterior insula) during fear acquisition and lower threat-regulating activity (vmPFC, hippocampus, dACC) during extinction and extinction recall. To test for specificity, we evaluated whether these associations (a) remain when the models include Anhedonia-Apprehension, (b) remain when accounting for reward-related brain activity, (c) are stronger than the associations between General Distress and Fears with neural response to reward, and (d) are specific to acquisition, extinction, or extinction recall.

## Materials and Methods

### Sampling and Participants

Participants were recruited from college campuses, community centers and community notice boards in Los Angeles and Chicago. Recruitment occurred as part of the ‘Brain, Motivation and Personality Development’ (BrainMAPD) study, a multi-site longitudinal project investigating reward and threat sensitivity in late adolescence to early adulthood, conducted at UCLA and Northwestern University (R01 MH100117). In this strategically crafted sample, participants were recruited based on self-reported trait Neuroticism using the Eysenck Personality Questionnaire-Neuroticism (EPQ-N) (32) and Reward Sensitivity using the Behavioral Activation Scale (BAS) (33) from among 2,461 individuals who completed screening. Participants were recruited to ensure sampling from high/mid/low ranges (tertiles) on both scales, with oversampling from the two diagonals of the bivariate space defined by the quasi-orthogonal EPQ-N and BAS scales (i.e., high EPQ-N/high BAS, low EPQ-N/low BAS, mid EPQ-N/mid BAS, high EPQ-N/low BAS and low EPQ-N/high BAS). This approach maximized variance in reward and threat sensitivity within our sample to ensure symptom diversity and given that variation in reward and threat sensitivity are associated with risk for mood and anxiety disorders (20,34). Participants were aged 18–19 years, as this is a period associated with heightened risk for internalizing psychopathology (30) and maturation of corticobasal ganglia and corticoamygdala circuitry (35).

Participants meeting initial screening criteria were excluded for left handedness, lack of English fluency, MRI contraindications, lifetime psychotic symptoms, bipolar disorder, substance use disorder in the past 6 months, and antipsychotic use. To maintain ecological validity, participants taking other psychoactive medications (N = 21) were retained.

The final sample included 339 participants (see Table 1), of whom 272 completed the fMRI protocol. Among the 272 participants who completed fMRI, data were set to missing for the following reasons. In the MID reward task, data from 49 participants were set to missing due to excessive motion or poor task performance (223 retained). In the Pavlovian fear learning task, excessive motion led to missing data for 43 participants during acquisition (229 retained), 54 during extinction (218 retained), and 61 during extinction recall (211 retained). However, we used full-information maximum likelihood to accommodate missing data, and thus the full sample of 339 participants was used for each analysis. Participants provided informed consent, and procedures were approved by the IRB at each institution.

Data from this sample have been published on previously. This study extends our prior work by modeling reward and threat neural activity within the same participants. We then examine, for the first time, whether neural correlates of the tri-level model of depression and anxiety are specific to, or shared across, reward and threat processes, as well as across broad symptom dimensions spanning both disorders.

### Measures

#### Multi-method tri-level dimensional symptom model measures.

Structural models of psychopathology, such as the tri-level model (36) and the Hierarchical Taxonomy of Psychopathology (HiTOP) (37), conceptualize mental disorders as dimensions of symptoms that capture shared and distinct variance across conditions rather than as discrete diagnostic categories. As in our prior work (25–27), we employed a hierarchical tri-level model with three levels: a broad general factor (General Distress), two intermediate factors (Fears and Anhedonia-Apprehension), and several narrow factors. As a hierarchical model, the tri-level model allows each item to load directly on multiple uncorrelated factors (38). We included both self-report indicators and interviewer-rated indicators of each factor (25). Self-report included items from the following scales: Fear Survey Schedule-II (39), Albany Panic and Phobia Questionnaire (40), Self-Consciousness subscale of the Social Phobia (41,42), Inventory to Diagnose Depression (43), Mood and Anxiety Symptom Questionnaire (44), Penn State Worry Questionnaire (45), and Obsessive Compulsive-Inventory Revised (46). Interviewer-ratings of symptom severity were extracted from the Structured Clinical Interview for DSM-5 (47) at T1-T4 (see 24 and Supplement). The factor scores generated through these procedures generated the symptom dimensions of General Distress, Fears, and Anhedonia-Apprehension in all analyses. Model fit was tested using Mplus (48).

Cross-sectional analyses were embedded in a multi-year longitudinal study. Participants completed the interview and self-report questionnaires assessing mood and anxiety symptoms noted above at baseline (T1) and again at 10, 20, and 30 months (T2-T4). T1 symptoms were assessed at the baseline MRI scan, which is the focus of the present paper. We also used symptom data from T2-T4 to identify the latent starting value (i.e., latent intercept) for General Distress, Fears, and Anhedonia-Apprehension, and these latent intercepts were used as the symptom data for all T1 cross-sectional analyses. The benefit of this approach is that estimates of T1 starting values or latent intercepts (i.e., the symptom data for this paper) account for measurement error and do not solely rely on observed T1 scores (see Supplement for additional details and Supplement Figure 1 for a schematic overview of this approach) (49).

At T1, 18% (n=61) of the 339 participants included in the final sample met criteria for a current anxiety disorder and 6% (n=21) for a current unipolar depressive disorder (with 4%, n=15, meeting criteria for both). Using an empirically derived cutoff, 29.1% of the sample scored within the clinical range on T1 General Distress (50), indicating substantial variability in symptom severity.

### Monetary Incentive Delay (MID) task

Participants completed the MID task to assess reward-related brain activity during anticipation and receipt of monetary rewards and losses (51). First, a circle cue signaling a reward trial (participant might Win $0.00, Win $1.50, or Win $5.00) or a square cue indicating a loss trial (participant might Lose $0.00, Lose $1.50, or Lose $5.00) was presented for 2 seconds. Then, a jittered fixation was followed by a solid white square. Participants were instructed to make a button response when the solid white square was on the screen to either win money (reward trials) or avoid losing money (loss trials; see Supplement for additional details). The anticipation period was the start of the cue signaling trial type with a 4 s duration. The outcome period was the start of the feedback with a 4 s duration.

### Pavlovian Fear Learning Task

Participants completed a differential Pavlovian fear conditioning task (49). Three phases occurred across two fMRI sessions: acquisition and extinction (session 1), and extinction recall (session 2). (see Supplement for details). Each trial involved a 3-sec context cue (background image), followed by a 6-sec conditional stimulus (CS; lamp light of different colors) embedded into the context. Acquisition involved one CS- and two CS+, with 62.5% co-terminating with a shock (US). Only one CS+ was extinguished (CS+E) and both CS+E and CS+U were tested at extinction recall. Galvanic skin conductance was recorded throughout. After acquisition and extinction, contingency awareness assessments examined whether participants correctly formed CS– US associations.

### MRI acquisition and processing

Data were acquired using a Siemens Prisma 3.0 T MRI scanner with a 64-channel head coil. Identical scanners and sequences were used at the two sites (see Supplement for details). Functional data were assessed for outlier volumes (75th percentile + 1.5-time interquartile range) based on framewise displacement. Data exceeding 10% outliers were set to missing for group analyses. fMRI data were processed with FEAT (FMRI Expert Analysis Tool) Version 6.00n using standard procedures. Outlier volumes were censored in first level analyses by including a regressor with a single time point corresponding to each outlying volume. We report the number of participants with data set to missing for motion or poor task performance in the Participant section above and the supplement. However, we used full-information maximum likelihood to accommodate missing data, and thus the full sample of 339 participants was used for each analysis.

#### MID processing:

**Figure 1.**
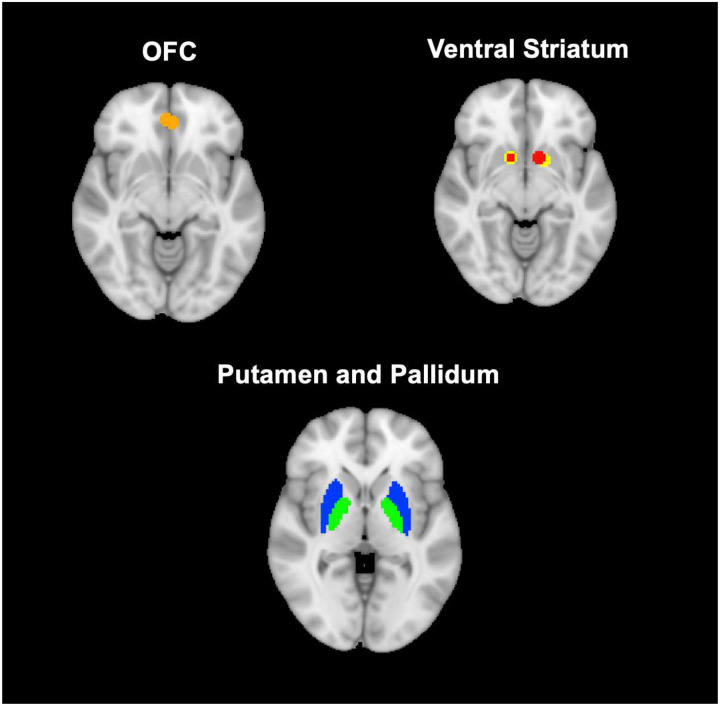
Region of Interests (ROIs) for monetary incentive delay (MID) reward task. Orbitofrontal cortex (OFC) = orange; ventral striatum during anticipation period = red; ventral striatum during outcome period = yellow; putamen = blue; pallidum = green.

First-level voxel-wise z-statistics were generated contrasting reward anticipation (i.e., Win $1.50, Win $5.00) vs nonreward (i.e., Win $0.00) and gain outcome vs no-gain (53). Guided by meta-analysis (54), 5-mm spheres were generated around peak activation coordinates for ventral striatal ROI analyses during anticipation and outcome. The same meta-analysis informed OFC ROIs during the outcome (54), as this region is not consistently activated during anticipation. Activation was averaged across three 5-mm spheres to compute a single OFC ROI. The Harvard-Oxford atlas defined ROIs for the pallidum and putamen (see Figure 1).

#### Pavlovian fear learning task processing:

**Figure 2.**
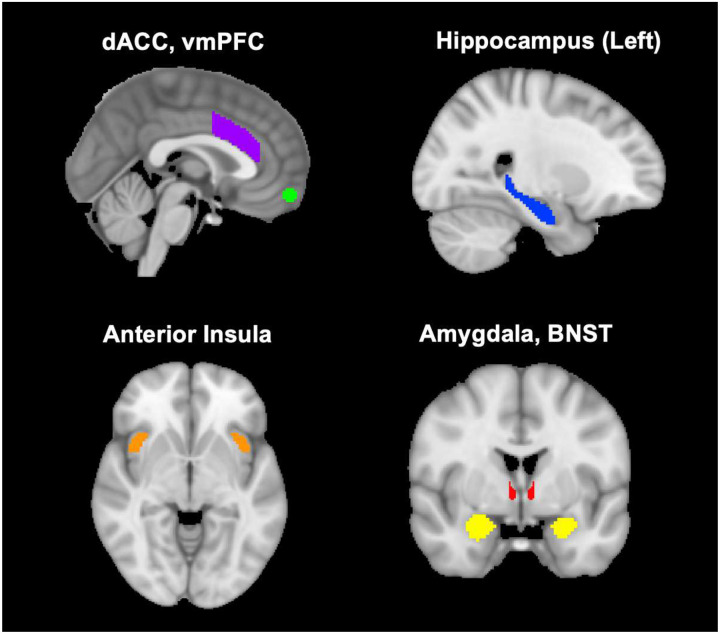
Region of Interests (ROIs) for Pavlovian fear learning task. Dorsal anterior cingulate cortex (dACC) = purple; ventromedial prefrontal cortex (vmPFC) = green; hippocampus = blue; anterior insula = orange; amygdala = yellow; bed nucleus of the stria terminalis (BNST).

First-level analyses included regressors of interest (acquisition: context, CS+(E/U), CS− and shock; extinction: context, CS+E, CS−; extinction recall: context, CS+E, CS+U, CS−), temporal derivatives, six motion regressors, and regressors to censor outlying volumes. The BNST ROI was anatomically defined as in prior research (55). The amygdala, anterior insula, dACC, and hippocampus ROIs were defined from the Harvard-Oxford atlas. The vmPFC was defined as a 5-mm sphere around peak activation from a meta-analysis of human fear conditioning (56) (see Figure 2). Analyses were conducted for each phase of fear learning using contrasts from prior fear conditioning literature (23,57,58): for acquisition “CS+ vs. CS −” (all trials), for extinction “late CS+ E vs. late CS−” (last four trials of each type), and for recall “early CS+ E vs. early CS+ U” (first four trials of each type).

### Data analysis plan

Analyses were conducted in M*plus* version 8.7 (59) using full-information maximum likelihood (FIML) to handle missing data (e.g., fMRI data either not collected or set to missing due to motion). Rather than excluding participants with incomplete data, FIML estimates model parameters using all available data from each individual. This approach allowed us to retain all 339 participants in each analysis. Because regression coefficients were estimated using maximum likelihood within a structural equation modeling framework, statistical tests are based on large-sample (asymptotic) normal theory and therefore do not involve degrees of freedom. Cutoffs for adequate fit for comparative fit index (CFI) values ≥ 0.9, root-mean-square error of approximation (RMSEA) values ≤ 0.06 and standardized root-mean-square (SRMR) values ≤ 0.08 (60) and were applied flexibly (60,61).

#### Symptom dimensions of anxiety and depression.

To generate factor scores of General Distress, Fears, and Anhedonia-Apprehension for cross-sectional analyses, we fit the multi-method version of the tri-level model at T1 (as reported in (24), fit was adequate: CFI = .93, RMSEA = .023, SRMR = .08). Next, we extracted factor score estimates of General Distress, Fears and Anhedonia-Apprehension at T2, T3 and T4 by specifying a version of the model at each subsequent wave that was scalar invariant in relation to the first wave (T1). We used these factor scores in latent growth curve models to extract the latent starting value (i.e., latent intercept) for General Distress, Fears and Anhedonia-Apprehension. These latent intercepts were used as the symptom data for T1 cross-sectional analyses and were the dependent variables in our primary analyses. The benefit of using the latent starting value as the symptom data is that accounts for measurement error (62).

#### Measurement models for fMRI variables.

We used a latent construct approach wherever justified by the data to manage multiple comparisons. fMRI latent variables and observed variables were modeled using ROIs from the first-level analyses of the MID reward and Pavlovian fear learning tasks (see Supplement for additional details. See Supplement Table 1 for correlations among the MID reward ROIs and Supplement Table 2 for correlations among the Pavlovian fear learning ROIs. See Supplement Table 3 for factor loadings for the MID reward ROIs and Supplement Table 4 for factor loadings for the Pavlovian fear learning ROIs). When a given ROI had both a left and right hemisphere, we averaged activation across hemispheres to minimize Type I error and because we did not have laterality hypotheses.

The final reward latent variable model included anticipation and outcome latent variables for reward generating ROIs (ventral striatum, putamen, pallidum) plus the observed OFC ROI during the outcome phase from the MID reward task. As discussed in the introduction, we expected to observe dissociable latent variables for threat-generating (amygdala, BNST, insula) and threat-regulating (vmPFC, dACC, hippocampus) ROIs from the Pavlovian fear learning task. This expectation was not supported by the data, and instead the final threat latent variable model combined threat-generating and threat-regulating ROIs into a single threat latent variable, separately for the acquisition, extinction and extinction-recall periods.

#### Regressions of Symptom Dimensions on Reward Generating Latent Variables and OFC activation (MID Reward Task) and on Threat Latent Variables (Pavlovian Fear Learning Task), and on Unique Variance in each ROI.

We conducted regressions for each symptom dimension (General Distress, Fears, Anhedonia-Apprehension) as the dependent variable: one with the reward-generating latent variable and OFC ROI as predictors and one with threat latent variables as predictors (see Supplement Tables 5 and 6 for correlations). Whenever there was a significant association between a reward or threat latent variable and a symptom dimension, we tested whether effects were driven by unique variance in each ROI. Further, if any ROI within a reward or threat latent variable was significantly correlated with a symptom dimension, but the corresponding latent variable was not associated with that symptom dimension, we tested whether the unique variance in that ROI was associated with the symptom latent intercept. This approach allowed us to leverage the benefits of using the reward and threat latent variables while also examining which individual brain regions show unique associations with symptoms.

#### Adjusting for potential covariates.

We considered site, sex and current psychotropic medication use (yes/no) as potential covariates by examining their associations with both the independent and dependent variables. Based on these analyses, current psychotropic medication use, and sex were covaried in analyses of General Distress and sex was covaried in analyses of Anhedonia-Apprehension (see Supplement for details).

#### Testing Specificity of Associations between Reward and Threat Variables and Symptom Dimensions.

For each significant association between a reward and threat variable and a symptom dimension, we tested specificity in four ways. First, we added the other two symptom dimensions as predictors (e.g., does the association between a threat latent variable and symptom dimension of Fears persist when General Distress and Anhedonia-Apprehension symptom dimensions are included?). Second, we added the other threat and reward variables (e.g., does the association between a threat latent variable and Fears symptom dimension persist when reward-generating latent variable and OFC activation during the MID Outcome phase are added?). Third, we evaluated if the association differed significantly from the associations with the other set of threat or reward variables (e.g., is the association between the Fears symptom dimension and the threat latent variable significantly stronger than its association with the reward generating variable?). Fourth, we tested whether the association differed significantly from associations with the remaining predictors within the same set of threat or reward variables. (e.g., if the Fears symptom dimension was associated with the threat latent variable during fear Acquisition, was that association significantly different from its associations with Extinction and Extinction Recall?).

## Results

### Associations of Symptom Dimensions with the Reward Generating Latent Variable and OFC Activation and the Unique Variance in each Reward ROI during the MID.

The symptom dimension Anhedonia-Apprehension was negatively associated with OFC activity during the Outcome period of the MID. (b = −.34, se = .147, p = .021). Because ventral striatal activity during the Reward Anticipation period showed a significant negative correlation with Anhedonia-Apprehension (Supplement Table 5), whereas the Reward Generating latent variable during Reward Anticipation was not significantly associated with Anhedonia-Apprehension (Supplement Table 7), we tested whether the unique variance in the ventral striatum during Reward Anticipation was associated with Anhedonia-Apprehension. The unique variance in the ventral striatum during Reward Anticipation was significantly negatively associated with Anhedonia-Apprehension (b = −.53, se = .218, p = .015). No other associations were significant between the reward-related variables and symptom dimensions (Supplement Table 7).

### Associations of Symptom Dimensions with Threat Latent Variables and the Unique Variance in each Threat Extinction ROI during the Fear Learning Task.

The symptom dimension Anhedonia-Apprehension was positively associated with the threat latent variable during Extinction (b = .21, se = .066, p = .001). None of the unique variances for individual ROIs within the threat latent variable during Extinction were associated with Anhedonia-Apprehension (Supplement Table 8). No other associations were significant between the threat-related variables and symptom dimensions (Supplement Table 9). Because the BNST during Extinction Recall (RBNST) showed a significant negative correlation (Supplement Table 6), whereas the Extinction Recall latent variable was not significantly associated with Anhedonia-Apprehension (Supplement Table 4), we tested whether the unique variance in the BNST during Extinction Recall was associated with Anhedonia-Apprehension. The unique variance in the BNST during Extinction Recall was not associated with Anhedonia-Apprehension (b = −.15, se = .099, p = .120).

### Adjusting for covariates.

None of the covariates altered the associations between symptom dimensions and the reward-generating latent variables or OFC activation during the MID reward task, or the threat latent variables during the Pavlovian fear-learning task.

### Specificity of Associations Between the Anhedonia-Apprehension Symptom Dimension and OFC Activation during the MID Outcome Period.

The negative association between the Anhedonia-Apprehension symptom dimension and OFC activation during the MID Outcome period remained significant and at least as large when General Distress and Fears symptom dimensions were entered in the model (b = −.40, se = .141, p = .005). It also remained significant and at least as large when the threat latent variables during the Pavlovian Fear Learning task were included in the model (b = −.35, se = .143, p = .014). This association was significantly more negative than the average effect of the threat latent variables during the Fear Learning task (Wald χ^2^(1) = 7.639, p = .0057). Finally, the average effect of the reward generating latent variables during the Anticipation and Outcome periods of the MID task did not differ from this association with OFC activation during the MID Outcome period. (Wald χ^2^(1) = 2.704, p = .1001).

### Specificity of the Associations between the Anhedonia-Apprehension Symptom Dimension and the Threat Latent Variable during Extinction in the Pavlovian Fear Learning Task.

The positive association between Anhedonia-Apprehension and the threat latent variable during Fear Extinction remained significant and at least as large (b = .25, se = .066, p = .000) when the General Distress and Fears symptom dimensions were entered in the model. It also remained significant and of similar magnitude (b = .21, se = .066, p = .001) when the reward-generating latent variable during the MID and OFC activation during the MID Outcome period were entered. The association between Anhedonia-Apprehension and the threat latent variable during Fear Extinction was significantly more positive than the association between Anhedonia-Apprehension and the average effect of all reward-related variables during the MID (Wald χ^2^(1) = 12.753, p = .0004). Finally, the average effect of the other two threat latent variables during the Fear Learning Task – Acquisition and Extinction Recall – was significantly less positive than this association with the threat latent variable during Fear Extinction (Wald χ^2^(1) = 5.171, p = .0230).

## Discussion

The present paper evaluated the specificity of associations between reward and threat-neural activity with comorbid symptoms of depression and anxiety. We assessed neural responses during reward- and threat-relevant tasks within the same participants and examined the specificity of their associations with symptom dimensions of negative affect shared between depression and anxiety (General Distress), low positive affect linked to depression (Anhedonia-Apprehension), and fears characteristic of phobias (Fears). We used a latent construct approach wherever justified by the data to manage multiple comparisons. For any significant association between a latent variable and a symptom latent intercept, we tested associations due to the unique variance in each ROI to disentangle whether specific subregions within each latent factor drove the observed effects. Current psychotropic medication use, and sex were covaried in analyses of General Distress, and sex was covaried in analyses of Anhedonia-Apprehension.

With respect to reward, we hypothesized that higher Anhedonia-Apprehension would be uniquely associated with lower reward-generating (ventral striatum, pallidum, putamen) and regulating (OFC) brain activity. The latent variable approach confirmed a latent reward-generating variable for both the anticipation and outcome phases of the MID task, with OFC during outcome serving as the sole cortical and regulatory reward ROI. As predicted, heightened Anhedonia-Apprehension was associated with lower OFC activation during the MID outcome phase. Specificity tests showed this association was specific to Anhedonia-Apprehension relative to other symptom dimensions, specific to OFC relative to the threat latent variable, and stronger than associations with the threat latent variable. This finding extends prior literature and highlights the unique relationship of low-reward related brain activity with anhedonia. Anhedonia-Apprehension was not associated with the reward generating latent variable for either MID phase. Anhedonia-Apprehension, however, was associated with unique variance in the ventral striatum. Specifically, higher Anhedonia-Apprehension was associated with lower ventral striatal activation during reward anticipation, which is consistent with prior research (63–65). These findings provide compelling evidence that heightened anhedonia is associated with lower activation in both the OFC and the ventral striatum during reward processing. None of the other brain areas in the reward generating latent variable were uniquely associated with Anhedonia-Apprehension.

With respect to threat, we hypothesized that both General Distress (common to anxiety and depression) and Fears (relatively specific to anxiety) would relate to heightened threat-generating activity (amygdala, BNST, anterior insula) during fear acquisition and lowered threat regulating brain activity (vmPFC, hippocampus, dACC) during extinction and extinction recall. Rather than separating threat-generating (amygdala, BNST, insula) and threat-regulating (vmPFC, dACC, hippocampus) ROIs from the Pavlovian fear learning task, our threat latent model combined the threat-generating and threat-regulating ROIs into a single threat latent variable, separately for the acquisition, extinction, and extinction-recall periods. Contrary to hypotheses, the only significant association was a positive relationship between Anhedonia-Apprehension and the latent threat variable during extinction. Specificity tests showed this association was specific to Anhedonia-Apprehension relative to other symptom dimensions, specific to threat relative to reward variables, stronger than associations with reward variables, and specific to extinction relative to acquisition and extinction recall. Further, this association was specific to the latent threat variable, and Anhedonia-Apprehension was not associated with the unique variance in any individual brain regions that comprised the latent threat variable.

Although contrary to hypotheses (19,20), our findings are consistent with some earlier work. Specifically, we previously observed that greater Anhedonia-Apprehension was associated with larger threat responses to the CS+ relative to the CS- at the end of extinction, indicative of weakened extinction (12). Moreover, a whole-brain MVPA decoder during extinction predicted Anhedonia-Apprehension and not General Distress or Fears (66). These associations may reflect reliance of fear extinction on dopaminergic “reward” pathways during prediction-error when an unexpected aversive US does not occur (67,68). Since anhedonia is associated with reward hyposensitivity (e.g.,63), higher Anhedonia-Apprehension may impair reward-dependent prediction error learning during extinction. That this relationship remained significant when reward variables were included in the model could mean that the way Anhedonia-Apprehension relates to reward processing during fear learning is different from reward processes measured during the MID. However, given these earlier reports involved the same sample of participants used in this study, future research with independent samples is needed to better understand the association between threat-related brain activity and anhedonia.

It is surprising that neither General Distress nor Fears related to threat-related brain activity during any phase of fear learning. One possibility is that the Pavlovian fear learning task did not induce a defensive state. Yet, GSR data confirmed acquisition and extinction of fears during the Pavlovian fear learning task (see Supplement). Nevertheless, our findings question whether anxiety reflects altered threat processing and raises the possibility that low positive affect or anhedonic features of anxiety (26,27,70) better explain the observed effects. Given the specificity with fear extinction, it may be that low positive affect is particularly relevant to fear persistence (i.e., resistance to extinction) rather than fear acquisition. Clearly, replication in other samples is needed.

The findings should be interpreted in light of several limitations. First, the cross-sectional design precludes causal inference. Longitudinal work is needed to determine whether alterations in threat and reward processing precede symptoms and whether they covary during developmental risk periods such as the transition to adulthood. Second, participants were college students and studies with more diverse samples are needed to replicate and extend these findings.

This study advances our understanding of relationships between threat and reward processing and empirically derived symptom dimensions of depression and anxiety. It extends work on depressive symptoms by showing that lower OFC and ventral striatal reward activity are uniquely associated with anhedonia-apprehension and underscores the need to further examine links between anhedonia and threat-related activity. More broadly, this work highlights the value of examining associations between specific mechanistic pathways (e.g., reward and threat processing) and symptom dimensions rather than categorical diagnoses. Such an approach may help address comorbidity, within-diagnosis heterogeneity, and inform more precise targeted interventions.

## Supplementary Material

Supplementary Files

This is a list of supplementary files associated with this preprint. Click to download.

- cNusslockBrainMAPDSupplementTPFinal060626.pdf

## Figures and Tables

**Table 1.** Demographic Variables

Sex (N, %)	
Female	227 (67.2%)
Male	110 (32.5%)
Transgender	1 (0.3%)
Age (M, SD)	19.86 (1.01)
Ethnicity	
Not Hispanic/Latino	251 (74.3%)
Hispanic/Latino	87 (25.7%)
Race	
White	177 (52.4%)
Asian	99 (29.3%)
Black	30 (8.9%)
Native American	6 (1.8%)
Multiracial	25 (7.4%)
Declined	1 (0.3%)
